# Chemo-Preventive Action of Resveratrol: Suppression of p53—A Molecular Targeting Approach

**DOI:** 10.3390/molecules26175325

**Published:** 2021-09-02

**Authors:** Rokeya Akter, Md. Habibur Rahman, Deepak Kaushik, Vineet Mittal, Diana Uivarosan, Aurelia Cristina Nechifor, Tapan Behl, Chenmala Karthika, Manuela Stoicescu, Mihai Alexandru Munteanu, Cristiana Bustea, Simona Bungau

**Affiliations:** 1 Department of Pharmacy, Jagannath University, Sadarghat, Dhaka 1100, Bangladesh; rokeyahabib94@gmail.com; 2 Department of Global Medical Science, Yonsei University Wonju College of Medicine, Yonsei University, Wonju 26426, Korea; 3 Department of Pharmacy, Southeast University, Banani, Dhaka 1213, Bangladesh; 4 Department of Pharmaceutical Sciences, Maharshi Dayanand University, Rohtak 124001, India; deepkaushik1977@gmail.com (D.K.); vineetmittalmdu@gmail.com (V.M.); 5 Department of Preclinical Disciplines, Faculty of Medicine and Pharmacy, University of Oradea, 410073 Oradea, Romania; diana.uivarosan@gmail.com; 6 Analytical Chemistry and Environmental Engineering Department, Polytechnic University of Bucharest, 011061 Bucharest, Romania; aureliacristinanechifor@gmail.com; 7 Department of Pharmacology, Chitkara College of Pharmacy, Chitkara University, Punjab 140401, India; tapanbehl31@gmail.com; 8 Department of Pharmaceutics, JSS College of Pharmacy, JSS Academy of Higher Education & Research, Ooty 643001, India; karthika1994haridas@gmail.com; 9 Department of Medical Disciplines, Faculty of Medicine and Pharmacy, University of Oradea, 410073 Oradea, Romania; manuela_stoicescu@yahoo.com (M.S.); mihaimunteanual@yahoo.com (M.A.M.); cristianabustea@yahoo.com (C.B.); 10 Department of Pharmacy, Faculty of Medicine and Pharmacy, University of Oradea, 410028 Oradea, Romania; 11 Doctoral School of Biological and Biomedical Sciences, University of Oradea, 410087 Oradea, Romania

**Keywords:** resveratrol, natural compound, polyphenol, red wine, p53, cancer prevention, cardio protection, molecular signals, clinical trials

## Abstract

Extensive experimental, clinical, and epidemiological evidence has explained and proven that products of natural origin are significantly important in preventing and/or ameliorating various disorders, including different types of cancer that researchers are extremely focused on. Among these studies on natural active substances, one can distinguish the emphasis on resveratrol and its properties, especially the potential anticancer role. Resveratrol is a natural product proven for its therapeutic activity, with remarkable anti-inflammatory properties. Various other benefits/actions have also been reported, such as cardioprotective, anti-ageing, antioxidant, etc. and its rapid digestion/absorption as well. This review aims to collect and present the latest published studies on resveratrol and its impact on cancer prevention, molecular signals (especially p53 protein participation), and its therapeutic prospects. The most recent information regarding the healing action of resveratrol is presented and concentrated to create an updated database focused on this topic presented above.

## 1. Introduction

New pieces of evidence are reported in demand with the use of natural compounds (such as polyphenols) for the treatment and mitigation of various disorders including cancer, a disease which involves abnormal proliferation of the body cells that, after being altered, could migrate to other organs than the one initially affected. This disorder is a large and rising cause of global death, with an estimated rise of 19.3 million new cases of cancer a year in 2025, and with more than one hundred different types of cancer known [1]. Cancer therapies include surgery, radiation, and clinical procedures that comprise cytotoxic chemotherapy, hormone treatment, antibody therapy, and many others [2].

Natural moieties or agents to prevent cancers of humans are becoming more widespread. Most epidemiological statistics suggest that the consumption of “unhealthy” types of food (i.e., sugary drinks; white bread; fried, grilled, or broiled food; pastries, cookies, and cakes; junk foods; low-fat milk products; processed meat and cheese; etc.) and the prevalence of several forms of cancers are closely associated. The growing trend of cancer from year to year around the world is certainly due to the modern lifestyle involving unhealthy eating habits—this being one of the main causes that induce, not only cancer, but also other disorders [3,4,5].

From the earliest times there have been many plants used by humankind as natural remedies in trying to cure and/or ameliorate certain diseases [6]. Over time, knowledge about plants has been enriched, as scientists have increasingly focused on the human body, its health, and methods of maintaining it (including diet). Additionally, they have intensified their attention on the biological compounds that can be capitalized and considered for human benefits, these types of substances proving to be more and more efficient in recent years. There are very encouraging results regarding the effects and role of natural compounds extracted from plants in better management of many diseases, including cancer. Published experimental, clinical, epidemiological reports, etc., have noted the anticancer properties of some plant-derived substances used in human diet. Micro-components that are found naturally in these plant-based foods provide benefits that are still insufficiently quantified for human health, resveratrol being such a compound—a natural polyphenol, it offers many medical options, challenges, and benefits, including rapid digestion, antioxidant proprieties, cardio protection, anti-diabetic and anti-ageing role, cancer prevention, and many others [7,8,9,10,11].

Resveratrol is chemically recognized as 3,4′,5-trihydroxy-trans-stilberene and has the Chemical Abstract Service (CAS) Registry Number 501-36-0. This compound can be found at its peak concentration in more than 70 plant species, especially in fruits (e.g., peanuts, berries—blueberries, mulberries, cranberries, and raspberries, and grapes, etc.) [7]. The efficient extraction of the resveratrol from grapes during the winemaking process is well known, red wine especially being proven to be one of the most important dietary source for this active component [8,11]. In this regard, it is worthy to mention an interesting study which has been published almost two decades ago, known as the French Paradox, on the moderate consumption of red wine that leads to lowering the cardiovascular disease evolution [9].

Since its discovery in 1997, resveratrol has been initially administered topically to mice for skin cancer, and the results of such experiments have been published over the years in several articles. It has also been found that extra doses of resveratrol given to animals protect them against all the harmful effects of fat-free diets and provide nutritional benefits [10,11,12,13,14]. As a natural compound, it has been tested for multiple disorders, including cancer prevention and treatment, being identified as a possible therapeutic agent [15].

Given that resveratrol is phytoalexin, with increased synthesis in response to phytopathogenic infection (with bacteria or fungi), the researchers also showed interest in exploring and exploiting its antimicrobial activity [16,17]. Moreover, resveratrol has gained consideration, being recognized for its role and for many biological activities (including on carcinogenesis) through the control of different signal transduction pathways.

Thus, considering all the above, the actuality, potential, and impact of the present topic is obvious. This review aims to collect and present both the most relevant and recent literature on resveratrol, as well as its impact on cancer prevention, molecular signals (especially p53 protein participation), and therapeutic perspectives that can be investigated. The summary information is a solid and highly informative database for those interested in this field related to resveratrol usage as a potential anti-cancer agent.

## 2. Methodology

To carry out this review, the authors have been involved in comprehensive research of the literature published on this theme, selecting those scientific papers that address resveratrol as chemical substance, resveratrol implications in cancers, pathways of action, clinical trials, etc., highlighting also the most relevant and interesting aspects. The publication interval of the selected articles was unlimited, being equally considered, including the articles published this year (until the submission date of the current study). The most well-known medical and biology databases (PubMed, Cochrane Library, Web of Science, etc.) have been accessed in order to obtain accurate and complete information. Just to give a first idea about the complexity of the work performed, when a search was undertaken on PubMed on the title “resveratrol” and “cancer”, 3975 search results were found on July 2021.

**Figure 1:**
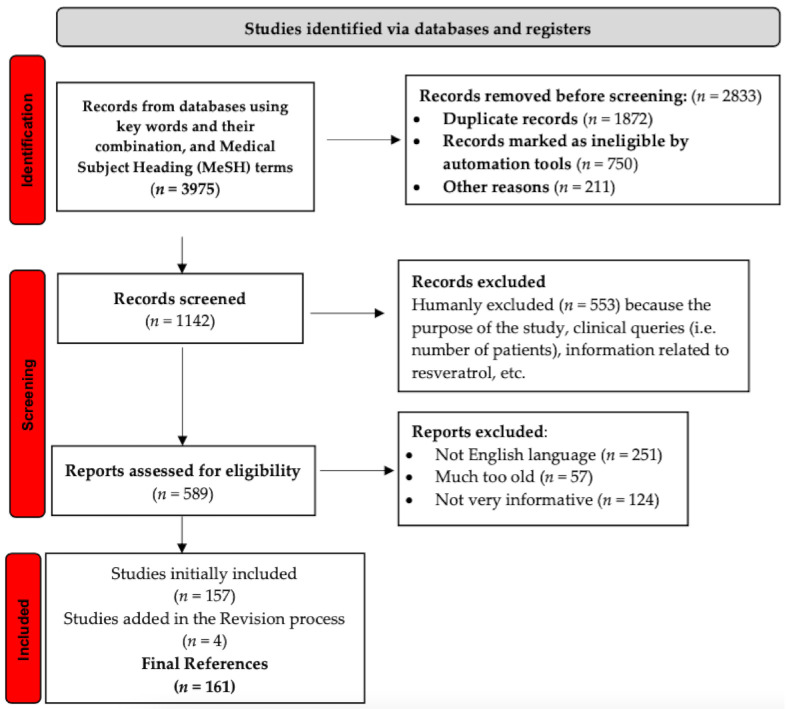
PRISMA flow-chart describing the process of selection the references.

The criteria further used to select the appropriate bibliography are summarized in Figure 1 (a PRISMA flow chart), which highlights the whole process extremely visibly and clearly, in the respect of Page et al.’s recommendations [18,19]. The keywords listed as being the most important at the beginning of this paper (resveratrol; natural compound; p53; cardio protection; cancer prevention; molecular signals; natural polyphenol; clinical trials; and others) and the Medical Subject Heading (MeSH) terms were applied to search for the most appropriate published data. The potential articles considered eligible were chosen first by their title, keywords, and abstract; then, the analysis of their content was decisive, this process being facilitated by filtering techniques (i.e., Clinical Queries). The most informative and relevant results and data were extracted, and the source was used as reference.

## 3. How Resveratrol Regulated Cell Signaling Pathways in Cancer

Resveratrol is proven to be involved in the carcinogen detoxification and procarcinogen bioactivation signaling pathway. Found to reduce oxidative stress, inflammation, and apoptosis induced through the activation of intrinsic and extrinsic pathways, it also manifests anticancer effect [10]. Resveratrol is reported as an active tumor growth inhibitor in various experimental models. Additionally, it is recognized for its increased ability to produce anti-angiogenetic effects, thus having potent metastatic potential on cancer cells [10].

The collected data indicate that various mediators have been able to modulate cancer initiation, promotion, and progression, as Jang et al. first reported the in vivo anti-tumor activity of resveratrol [10]. Numerous ways to prevent, interrupt, or delay tumor growth were consequently suggested [20,21,22]. The cytochrome P450 enzymes present with reports of phase I and II (both in vitro and in vivo) was evaluated for the conformation of tumor activation repressor [23,24,25]. Genes such as *Cyp1A1, CYP1B,* and *CYP1A2* block the activation of transcriptional *CyP*, thus preventing the transformation of carcinogenic agents into potential carcinogens (i.e., the transcription of xenobiotic enzymes stage I) [25,26]. In addition to phase II enzymes, the development or functioning (such as in the case of glutathione peroxidase, glutathione s-transferase, uridine 5′-diphospho-glucuronyl transferase (UGT), nitrite reductase (NAD(P)H), etc.), has also been evaluated for its role [27].

Moreover, resveratrol controls some of the mechanisms and signaling channels, including those of procarcinogen bioactivation and carcinogenic detoxification, minimizing oxidative stress and inflammation, inducing apoptosis by stimulating external and subtle mechanisms resulting in cancer [28,29,30,31,32,33]. Resveratrol affects the three stages of carcinogenesis (tumor development, advancement, and progression) and prevents the final carcinogenesis steps, such as angiogenesis and metastases [34]. It also impacts mitochondrial functions, including tumor suppressor protein respiratory tract, oncoproteins, gene expression, etc., specifically associated with the p53 [35].

Additionally, in various cancers resveratrol is considered similar to a chemical sensitizer that reduces the cell death activation threshold, being a traditional antifungal agent and regulating tumor cell chemical resistance [36,37]. Owing to the estrogenic feature, resveratrol has some effects due to the intrinsic similarities with the synthetic estrogen diethylstilbestrol. It can bind, act as an agonist or as an anti-estrogen related receptor gamma antibody (targeting estrogen receptor concentrations, competition, and expression), sometimes causing opposite reactions, when resveratrol can act as a super agonist (i.e., in human MCF-7 cells) [38].

Many studies revealed the role of resveratrol in the intracellular redox state. This micro component, as all other polyphenols, acts as a key cellular antioxidant by defining cell concentration and form. Resveratrol has, however, been suggested as a prooxidant, to produce the anti-influenza effects of tumors in the lung. Resveratrol also reduces the ability of the mitochondrial membrane and increases reactive oxygen (ROS) production, thereby stimulating apoptosis [39,40]. In various animal models, resveratrol is known as an important inhibitor of tumor development. Past experiments have shown that cancers affect a variety of cultivated cells, such as colon, breast, lung, and leukemia [41,42,43,44,45,46,47,48]. Resveratrol works more effectively in the melanoma cell lines MDA-MB-435, inhibiting cell fraction decrease in the phase G1, and the associated accumulation of the cell in phase S. Additionally, several other published data have documented the resveratrol action on different human cells, in a micromolar concentration arrest in G1/S, phase S or G2/M [49,50].

Although some experiments have shown that resveratrol causes cell cycles through a reversible mechanism and does not cause apoptosis, several other findings have suggested that apoptotic cell death can be considered a follow-up method [51]. However, resveratrol has significant anti-angiogenic properties, which help to reduce the metastatic ability of tumor cells. The following pathways include inhibition of the extracellular matrix of the metalloproteinase gene expression of tumor invasively involving matrix metalloproteinases (MMP-2 and MMP-9), inhibition of the development of hypoxia-inducible factor 1α (HIF-1α), and vascular endothelial growth factor (VEGF), all these aforementioned factors being closely related to the formation of a new blood vessel [50]. While the activation of extracellular receptors has already demonstrated that certain noncarcinogenic effects of resveratrol are activated, there are some evidential reports that the cell-based internalization is needed to activate such intracellular targets. Multiphoton microscopy showed that resveratrol is effective on neuroblastoma cell through glucose metabolites, which allow the molecule to have anti-tumor effects, as opposed to other metabolites [52,53]. In vitro and cell culture research have found that resveratrol has pro-apoptotic potential, a model study by the xenograft revealing that this compound may inhibit tumor growth if administered orally [52,53].

Tumor growth inhibition may explain other mechanisms that have no pro-apoptotic effect, including proliferation and anti-angiogenic activity of resveratrol [54]. Studies based on the impact of resveratrol on the spontaneous carcinogenesis model in vivo are also minimal and conflicting. The supplements with resveratrol have shown favorable, neutral, and negative effects in these experimental studies, based on the route of administration, dosage, tumor size, species, and molecular properties of the type of cancer cell [55,56]. Thanks to these pleiotropic results, scientists find resveratrol as a potential anti-cancer medication and have focused their efforts on a detailed understanding of its action mechanisms.

## 4. Resveratrol and p53 Suppression

Known as an important protein that suppresses tumors, p53 also plays a central function in the prevention of cancer. Wild type p53 prevents the development of tumors through cell cycle inhibition, and/or apoptosis, p53 having the ability to control the transcription and the increasing arrest, cell division, and/or death of specific target genes involved in these processes (such as damage to the deoxyribonucleic acid (DNA), onco-genetic initiation, hypoxia, and telomere damage) [57,58,59]. Furthermore, p53 modulates cell death pathways through processes acting on the transcription factor of their activities or those which are independent of them. The transcription of this target gene is used for p53-mediated apoptosis, while p53-independent apoptosis is primarily associated with the antiapoptotic or proapoptotic proteins. Several studies have demonstrated the action of p53 with apoptotic pathways internally and externally impaired protein expression [60]. In reaction to p53, mitochondrial proteins (such as Noxa, PUMA, and p53AIP1) have shown increased expression. As well, p53 induces a transcription of the pro-apoptotic family of genes *Bcl-2* (such as *BAX* and *BAK)*, releases cytochrome-c to the cytoplasm and coordinates its connection to the apoptotic protease activation factor (Apaf-1). The network is qualified for caspase 9 (*CASP9* gene); in consequence, the caspase for drivers is disabled.

p53 can also promote apoptosis by active death receptors (such as Fas, DR4, and DR5) [61]. The route p53 is particularly vulnerable to small DNA damage, being essential for early tumor genetic injury diagnosis [62]. In response to this disruption, the activity of other proteins responsible for p53 activation (such as the checkpoint kinase 2 (Chk2)) is instead increased. Chk2 is a serine/threonine phosphorylation kinase that can be activated with the serine 20 residue that inhibits p53 mediated degeneration of mouse double minute 2 homolog (MDM2), thereby making it possible to stabilize the serine 20 residue via phosphorylation [63]. Stabilization of p53 helps to activate *Cip1*, a key target gene (a protein p21 inhibitor of the cycling-related kinase (CDK) used in the G1 cycle process). The CDKs allow the transformation from G1 to S and G2 to M, facilitating the synthesis and replication of divided DNA and cell. Inhibition is realized by stopping several inflammatory proteins and by preventing the progression of the cell cycle [64]. The enhancement of p53 by resveratrol induced by mitogen activated protein (MAP) kinases and the apoptosis process was noted. For the first time, researchers have demonstrated that resveratrol can increase endogenous levels of p53 in epidermal JB6 cells, particularly in phosphorylated conditions, these representing a well-developed cell culture model to study tumor development.

Interestingly, according to the presence of resveratrol, the levels of phosphorylated protein kinases (ERK, p38, and JNK) are growing over time [65,66]. Studies focused on MCF-7 cells suggested a mechanism that stimulates resveratrol by extracellular signal-regulated kinases (ERKs), activates p53 protein phosphorylate in turn, and is linked to plasma membrane integrity [53]. Evidence has shown that resveratrol can lead to p53-based death [67,68,69] in many cell lines. Earlier studies have shown that this micro component supports the cells expressing p53 rates by inducing post-translation changes, including phosphorylation and acetylation, to trigger and stabilize the tumor cell culture [67,68,69].

These above mentioned changes are necessary to transcriptionally activate p53-compliant genes [70]. Ironically, resveratrol and other polyphenolic compounds, irrespective of cell state p53, may also induce apoptosis. Recent studies have identified using alternative approaches including p73, a p53-like tumor suppressor [71]. Further research has shown that in the breast cells with resveratrol-induced apoptosis p53-dependent, independent pathways can cause cell death in wild-type p53-type cells, but not in protein-mutant-expressing cells [72]. Antiproliferative and pro-apoptotic resveratrol activities were demonstrated to have p53 regulated in cancer cells derived from the lung (A5,49), liver (HepG2), thyroid (FTC 236 and FTC 238), and osteosarcoma (SYSA1), etc. [73,74,75]. Resveratrol enhanced expression of p53-p(ser15) and/or p53-ac(lys 382) and augmented p53 protein without a change in p53 mRNA in prostate cancer cells. This compound has also been associated with the transition of mitochondria p53 and the change in the cell cycle [76,77,78,79]. It has also been revealed that hot spot mutants seem to be easier to combine than wild type p53. The amyloid origin of aggregates with various techniques were also shown in [76,77,78,79].

**Figure 2:**
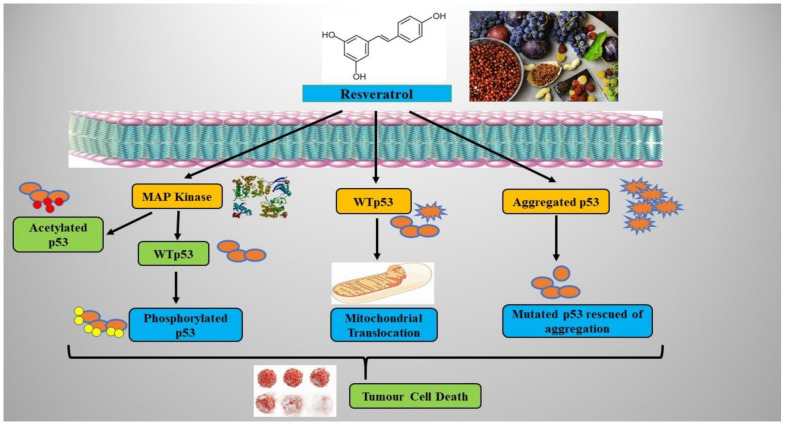
The contribution of p53 in the effects of resveratrol in cancer cells. MAP kinase, mitogen activated protein kinase; WTp53, wild type p53.

The introduction of wild type p53, prion forms, was shown to be the explanation for p53 R248Q mutant oligomers and fibrils. In lines of breast cancer, the co-location of p53 and aggregates have been found. MDA-MB231 cells show a significant increase in the cell nucleus of R280K p53 mutants with p53 aggregates [80]. Mutant p53 has also been recognized as the coaggregation of other proteins and can contribute to a gain of function phenotype. Furthermore, mutants p53, p63, and p73 appear to be present with their paralogs [81,82]. Amyloid p53 aggregate has also been found in many kinds of malignant tumors, of skin and ovarian cancer [83,84]. The bio-important behavior of p53 mutants was shown, and new strategies to disrupt aggregate formation were determined [85,86,87]. Resveratrol was found to prevent amyloid aggregation by bonding with various amyloid proteins including transthyretin, islet amyloid polypeptide (IAPP), and alpha-synuclein [88,89,90,91,92,93]. Published data examined the relationship between resveratrol and p53 and evaluated their effect on amyloid p53 [94,95]. Results showed that resveratrol could help inhibit part of tumor p53 aggregation. Such findings indicate that p53 pathways are involved in the effects of resveratrol in cancer cells, as presented in Figure 2.

## 5. Resveratrol and Its Bioavailability

Low bioavailability and a high metabolism of resveratrol are the main therapeutic problems persisting while dealing with the in vitro concentration, also being essential for many in vitro performed studies [96].

The bioavailability of ingesting and intravenous doses of resveratrol cannot exceed pharmacologically active plasma concentration due to their rapid phase II metabolism in the liver and intestines, but recirculating inter hepatically can lead to a delayed body removal and an extended impact. Resveratrol also has a prolonged effect by binding with plasma proteins [37,97]. Current plasma and artery absorption kinetics depend strongly on certain nutritional compounds. After obtaining pure resveratrol in high concentrations in wine or in diets rich in this compound, a boost of its bioavailability was observed after administration [98]. The average plasma growth during the administration of 300 mL red wine per day was determined for 15 days by Pignatelli et al. [99]. However, in these studies, the exact plasma level of resveratrol was not established, but it was revealed that the average level of resveratrol in the red wine experimentally used had a value of 8.2 μM [100]. Likewise, with a portion of the grape extract consisting of resveratrol (as part of the red wine metabolic profile and grape extract), the absorption and extension in the gut were decreased and prolonged [101]. About 25 mg of resveratrol results in free plasma concentrations of 1 to 5 ng/mL, higher doses of resveratrol produced free levels up to or just above 2 μM of approximately 500 ng/mL [102,103].

Another confounding aspect of resveratrol levels and metabolites in the body is the metabolism of the microbiota in human resveratrol. When human fecal microbiomes were used, a different metabolite profile was identified, so that gastrointestinal microbiota metabolism is also important. Published data have provided valuable insights into the molecular system, and their evidence support the assumption that the bio-effects of resveratrol can be transmitted by metabolites despite their lower in vivo bio-responsibility [104,105]. When studying antioxidants as well as their potential cytostatic effects, resveratrol was combined with specific stilbenes and demonstrated synergism (with pterostilbene and polydatin) [106,107]. In a variety of in vitro models, curcumin and resveratrol have been studied and antioxidant, cytostatic and apoptosis induction have also been evaluated [108].

Some flavonoids, including chrysin, quercetin, catechin, genistein, as well as several other combinations of flavonoids, have also been evaluated in combination with resveratrol. The results proved the dependence on the availability and variations of the studied compounds. Studies have usually shown that the same results were obtained after using mixtures of compounds, but in lower concentrations [109]. Ethanol also has a potentially significant effect on polyphenols solubility and cell absorption fluidity, while a high degree of ethanol counteracts the pharmacological profile of polyphenols [110].

Various studies have identified other polyphenols in red wine as chemical control agents (i.e., quercetin, catechin, and gallic acid) [111,112,113]. Moreover, it has been revealed that synergistic combinations of polyphenol extracts from grapes (in the form of, but not limited to wine) show increased anti-proliferation activity for the colon cancer cells [114].

Chemotherapeutic drugs are widely used to interlink DNA with rapidly developing cells such as cisplatin, carboplatin, and oxaliplatin. In most studies, a synergistic effect on cell viability (with respect to different cancer cell lines) was observed, at a time producing an additive effect [115,116]. Special effects of fluorouracil, fludarabine, cladribine, gemcitabine, clofarabine, etc. have been recorded by intercalating DNA agents, including doxorubicin and docetaxel, topoisomerase inhibitors, and analog nucleotides [117,118,119]. Combining the effect of the drug alone, resveratrol and DNA-alkylating compounds (cyclophosphamide, temozolomide, melphalan, and carmustine) contributed to potential results [120]. Resveratrol also demonstrated diminished or counteracted effects, depending on the course of treatment, when combined with microtubule inhibitors (vinblastine and paclitaxel) [120].

Styrene production and chemical synthesis have also been concerned with the identification of highly active molecules, especially cell proliferation inhibition, for medical applications [121]. Hydroxylation shifts and the methoxylation pattern of resveratrol had inhibitor effects on the SW480 (cell line) human colorectal tumor and did not affect non-tumor cells [122].

Resveratrol can interact with other polyphenols in an additive or synergistic way, and can affect other drugs’ behavior and metabolism. The synergies between various polyphenols and resveratrol were investigated and the results of other nutraceutical formulations were subordinated [123,124,125]. This challenge includes promising approaches such as quercetin or other flavonoids using natural or synthetic analogs that boost or have a higher potential than resveratrol and combine synergistic or bioavailable drugs [126]. As an anti-cancer drug, the latter strategy is very appealing as the drugs tend to lower doses of individual compounds leading to a greater therapeutic action as a result of additives and synergies and less side effects [127,128]. The bioavailability of nano encapsulated resveratrol for the parent compound was also enhanced [129,130]. Encapsulation and the use of alternate routes were studied [131].

## 6. Combined Synergistic Effects of Polyphenol Management

Synergistic therapeutic effects occur or manifest themselves using a combination of (at least) two polyphenols. For example, two polyphenols combined (resveratrol and quercetin) substantially encourage the percentage of senescent cells in cultured glioma cells than through single use [124]. The anti-cancer effects of traditional chemical treatments and therapeutics have also been enhanced by polyphenols. Resveratrol enhanced temozolomide induced senescence and further lowered glioma cell temozolomide resistance [132]. Additionally, resveratrol showed that the cancer cell resistance to paclitaxel can be re-sensitized and the gefitinib resistance of non-small cells of the lungs can be overcome by increasing the fitting intracellular concentration when used along with gefitinib [133].

**Figure 3:**
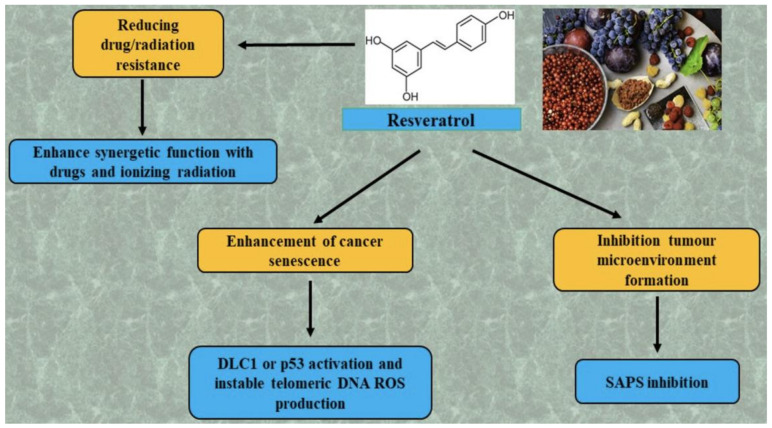
Potential anti-tumor effects of resveratrol. SASP, senescence-associated secretory phenotype; DLC1, deleted in liver cancer1; and ROS, reactive oxygen species.

The treatment with resveratrol also led to more DNA breakdowns and production of reactive oxygen species (ROS), which enriched ionized radiation premature senescence and reduced radio resistance in lung cancer cells [134]. The combination scheme resistant to doxorubicin has allowed doxorubicin-resistant gastric cancer cells to recover their sensitivity to the drug, reverse an epithelial-mesenchymal transition, and promote apoptosis in cells in vitro [135]. Eleven of these cases involve the polyphenols’ brilliant role in medicinal/radioresistant stubborn cancers. Potential anti-tumor effects of resveratrol are summarized in Figure 3.

The polyphenolic compounds are commonly included in the human diet, and recent results suggest that neurofibromatosis 1 has been successful in the treatment of patients by combining turmeric and the Medellan diet, a dietary pattern rich in polyphenols [136]. Combination therapy with resveratrol and other polyphenols may enhance the effects of the treatment applied to the patient, and may decrease the resistance of cancer cells while increasing the effectiveness of clinical therapy with radiation or drugs.

## 7. Resveratrol Clinical Trials and Therapeutic Potential

Note that the resveratrol doses used in the cell cultures and the in vivo concentrations are often distinguished. For example, various studies showed that resveratrol has a signaling effect of 10 μM to 100 μM on used concentrations [95]. On the other hand, the median plasma level of resveratrol was just 25 mg of resveratrol equal to high rotten wines and 2.4 μM in humans, with a 5 g transition dose [137]. When lower levels are added in cells, the outcomes are unpredictable, often without any effects [138]. Particularly, 2–3 orders of magnitude exceed the dietary doses of resveratrol in supplements are also available in various trials [139]. The plasma levels are 2–18 μM with one dose of resveratrol [140]. Eight weeks of intake of 3 g daily in obese people have led to fast and extensive resveratrol conjugation [141]. The research duration ranges from acute exposure to several days of exposure up to one year [14,142,143]. Resveratrol is given to people for a period of 1–3 months, in most trials. The relatively short duration of the trials raises some difficulties, as therapeutic, but not preventive, studies are needed. Research to prove that resveratrol is preventative should be performed for at least one year without taking account of practical and economic issues.

These trials are costly and not readily funded, but would gain useful information about the preventative/therapeutic potential of resveratrol [138]. Resveratrol studies in people with single/multiple daily doses (of up to 600 mg/day for two to three days) have demonstrated that resveratrol is safe in the test conditions; it was also revealed that the most important metabolites in the circulation of resveratrol are R3S, R4 G, and R3 G with especially high levels of sulfur conjugation [137,144,145]. Before 2010, several phase 1 clinical studies focusing on pharmacokinetics were conducted, but since then the number of clinical studies that have examined the bio-effect of resveratrol has substantially increased. It is recognized the fact that resveratrol is effective in human medicine, in recent years, for several pharmacological properties [146]. Clinical trials below briefly describe the main findings and results regarding the potential of resveratrol in cancer.

### 7.1. Clinical Trial 1

Resveratrol for patients with colon cancer: The first clinical trial for resveratrol in cancer patients is a study of Nguyen’s on biomarkers, investigating the expression of different components and target genes in a *Wnt* way. Two patients received resveratrol in doses of 20 mg/day, 80 mg/day in 3rd and 4th day, and 160 mg/day in 5th and 6th day. All the patients were given mixed extracts 125 mg/day, and an 8 oz glass of wine. No dose adjustments were made to avoid side effects associated with resveratrol in patients. As a result, *Wnt* genes, D1 cycling, and axin expression, combined with other bioactive ingredients, in the normal colonic mucosa, with a small dose of resveratrol, were inhibited, which shows that resveratrol-related inhibition of *Wnt* pathway can help prevent colon cancer [147,148].

### 7.2. Clinical Trial 2

A biological study of resveratrol effects: Winslow of the University of Wisconsin, Madison, performed a biological study on the effect of resveratrol on topics of low-level gastrointestinal cancer. The activation of *Notch-1*, which prevents the growth of tumor cells, is shown to be determined by resveratrol. In this study, the effects of resveratrol and *Notch-1* on tumor tissue and the tolerances of persons taking resveratrol for up to three months with neuroendocrine tumors (5 g a day, administered orally in two doses of 2.5 g each, with a minimal limiting dose according to the standard toxicity criteria of the National Cancer Institute) were investigated. Concentrations of the tumor markers were compared with post-treatment concentrations (e.g., chromogranin, 5-HIAA, gastrin). The response of the tumor was determined every 3 months. Additionally, the standard Response Assessment Criteria for Solid Tumors (RECIST) would be used for recording the response levels of the tumor.

### 7.3. Clinical Trial 3

Resveratrol and human hepatocyte function in cancer: The phase I bio-marker analysis was carried out by Randall Holcombe of the University of California, Irvine. The study aimed to determine the minimum levels of fresh red resveratrol-rich grapes needed to show signs of colon cancer prevention. The grape diet provided a low dose of resveratrol in combination with other potentially active ingredients in grapes. Small flexible sigmoidoscopies of colon tissue were collected before and after the ingestion of the red grape diet. Various doses have been received (1 or 2/3 or 1/3 lb/day of red fresh grapes). This work on *Wnt* biomarkers and essential data on the efficacy of this dietary strategy in the management of colon cancer has demonstrated the effects of low dose resveratrol extracted from grapes.

### 7.4. Clinical Trial 4

Biomarker study for resveratrol in colon cancer prevention: The randomized, two-blind, placebo-controlled, inpatient and outpatient trials are results from “a clinical trial in which health, drug kinetics and drug pharmacodynamics of SRT501 are assessed in subjects with colorectal cancer and hepatic metastases.” In patients with colorectal cancer scheduled for hepatectomy, micronized resveratrol (SRT501) was administered in a 5 g daily intake for 14 days. Micronization permits greater absorption of resveratrol, thus increasing the availability of SRT501. Mean plasma resveratrol levels were 1.9 ± 1.4 ng/mL at a single dose of SRT501, above the recorded levels of non-mice resistant resveratrol at equivalent doses 2- to 3.5-fold [15]. Resveratrol was seen in hepatic tissue after administration of SRT501. In malignant hepatitis, the precursor of apoptosis caspase-3 was significantly increased by about 39% in comparison with the tissue of placebo-treated patients following SRT501 [149].

### 7.5. Clinical Trial 5

Treating post-surgery patients: Pilot research on resveratrol in poor glucose tolerance elderly adults. The study analyzed its effect on post-meal glycemic metabolism. This proposed pilot research recommended the administration of resveratrol (as treatment: 500 mg capsules (totally 1500 mg) orally, twice a day, for six weeks) in old adults, suggesting that it will reduce tolerance [150].

### 7.6. Clinical Trial 6

Prevention in healthy patients: The study aims to explore the impact of different doses of resveratrol (75 mg or 150 mg/day on the health of the heart and blood vessels). Results suggested cardiovascular and restorative safety in elderly generations. This resVida study phase 1/2 took place over 12 months, in 90 overweight/obese people over 50 years of age (30 in each group) [151].

### 7.7. Clinical Trial 7

Pharmacokinetic and pharmacodynamic parameters metabolic syndrome and resveratrol: In order to enhance the symptoms of metabolic syndrome, the researchers considered to confirm resveratrol effects after being administered under controlled weight stability conditions, standard diet, and strict observance of treatment; it decreases the risk of diabetes and heart disease [152].

### 7.8. Clinical Trial 8

Healthy adults: Phase 1 studies did not support nephrotoxicity in metastatic colorectal patients. As SRT501 is highly metabolic, many myeloma patients are at risk for renal failure due to many factors. A total of 24 patients were enrolled in phase 2, chronic myeloma clinical trials with recurred patients, with/without bortezomib and 5 g SRT501, or with a minimum of one prior refractory therapy. For patients included in the research, the activity and safety of SRT501 alone or combined with bortezomib were evaluated [149,153]. Bortezomib stabilization may have avoided renal failure, but inadequate effectiveness (with nausea and vomiting) may cause dehydration and advanced renal failure. Renal toxicity was observed in 5 out of 24 patients. Renal dysfunction is one of multiple myeloma clinical signs and it has been identified in nearly half of the patients with the disease. For patients who have recurred/refractory multiple myeloma, this study has shown an unacceptable health profile, poor efficacy, and the risks of new drug production [154].

### 7.9. Clinical Trial 9

Postmenopausal woman: Resveratrol could potentiate inhibition of cell proliferation based on simvastatin and inhibit the mevalonate cycle, indicating new action mechanisms and emphasizing the potential translation and clinical significance of this micro component interactions with simvastatin [142]. A phase 4 clinical assessment is being carried out in Poland, on the effects of simvastatin and micronized trans-resveratrol on polycystic ovarian syndrome (PCOS). The effects of simvastatin (20 mg daily) and micronized trans-resveratrol on polycystic ovary syndrome are studied in both endocrine and metabolic disorders. Assessments take place based on the therapy lasting three to six months, and the key effect is an improvement in serum total testosterone and strong insulin levels. Dr. Frank González, principal investigator, Director of Endocrinology and Infertility Division at Indiana University, believes that the use of human chorionic gonadotropin (HCG) in females with PCOS induces excess ovarian androgens, that dairy cream intake enhances the activation of the white cell, and that the HCG-similar relationship occurs [142]. For three years, thirty women with PCOS (10 average weight, 10 overweights, with increased abdominal adiposity, 10 obese), and 30 women with ovulatory inspection (10 average, usually abdominal, 10 overweight, and 10 obese) were observed. The researcher also believes that the ovarian androgenic responses to HCG and inflammation responses to the consumption of dairy cream will be reduced, the adiposity reduced, the sensitivity to the insulin increased and the ovulation of average weight women with PCOS decreased over 12 weeks. Salsalate was given in a group of 16 women, 8 with PCOS (four with usual adiposes in the abdomen and four with increased abdomen adiposity) and 8 PCE (four with regular adiposity in the abdominal area, four with normal adiposities in the abdomen) for 3 years. This pilot project helps to decide whether a massive, double-blind, randomized study of PCOS women will further test the above hypothesis [142].

**Figure 4:**
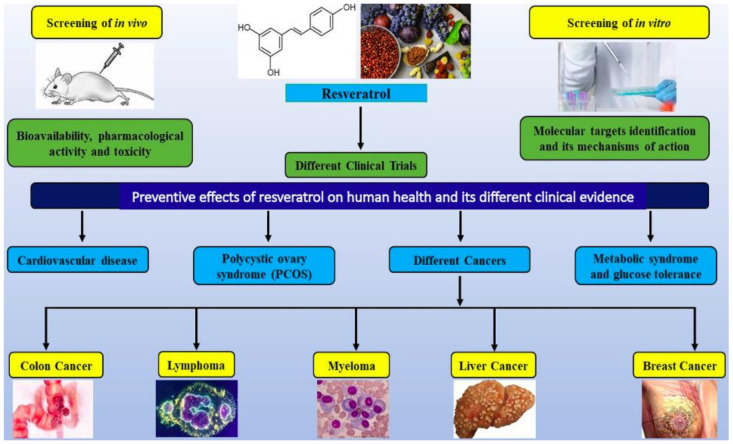
The preventive effect of resveratrol on human health in vitro, in vivo, and clinical evidence.

Figure 4 summarizes the clinical evidence of resveratrol preventive effects on human health, especially in cancer. Only 10 of them (50 percent) were completed based on the above reports.

The primary aim is to assess the dosage and side effects in clinical trials, in step 1. Eight of the clinical trials relate to various cancer types and show a preventive action of resveratrol. Furthermore, we suggest improving the quality of clinical trials for prospective patients through the appropriate designs, new formulations, and/or routes of administration, and biomarkers. Unfortunately, currently, the prevention and therapeutic effect of resveratrol only occur in vitro and in vivo model organism studies.

## 8. Adverse Effects

Resveratrol is normally well-tolerated, based on animal tests, and very few trials have been carried out in human cases of short or acute exposures. If eight healthy people were exposed to resveratrol twice a day for 8 days, six in eight people presented mild diarrhea, typically in the early part of the 8-day therapy period [155]. For the randomized, placebo-controlled analysis, two adult patients (men and women) healthy volunteers per group of 25, 50, or 150, received up to six times a day, 975 mg/day. Both groups had moderate and related adverse effects. Relatively high doses and a short period of administration have been used for resveratrol over time, however, relatively low plasma resveratrol levels have been produced [102]. Treatment with up to 270 mg resveratrol to 19 volunteers for one week did not cause pain [156].

In an exposure check of seven days, Elliott et al. reported that healthy participants tolerated resveratrol well, but experimental specifics were not presented and results were difficult to determine [157]. In the same article, a study with daily administration of 2.5 g or 5 g resveratrol for 28 days was briefly mentioned. The writers reported the general nature and reversibility of adverse events but did not provide experimental detail for a closer assessment [157]. In 20 patients receiving 0.5 g or 1.0 g resveratrol daily, there was strong tolerance eight days before the procedure [158].

## 9. Recommendations, Challenges, Future Perspectives, and Concluding Remarks

The first international conference on resveratrol and health in Denmark in September 2010, Resveratrol 2010, assessed the existing knowledge on the topic and offered guidance for usage and potential resveratrol studies. One of the main subjects at the congress was that numerous ways of determining whether the dosage or biomarker could characterize the drug may lead to the beneficial effects of resveratrol, making it difficult to decide [159,160]. Resveratrol 2012 (2nd edition of the International Conference on Health and Resveratrol) found that the proof submitted for clinical studies is not adequate to support the chronic clinical resveratrol intake recommendation at the University of Leicester, England. New animal data and new clinical trials are promising, and demonstrate the need for future research. Longer exposure to resveratrol has been suggested in new trials during the third International Resveratrol and Safety Conference, in Hawaii, 2014. No true clinical trials have been published to date investigating resveratrol in cancer [161].

Cancer refers to a condition characterized mainly by uncontrolled cell growth. The diagnosis of cancer is the main challenge for doctors in the field. If the diagnosis is late, classifying the cancer as being in the fourth stage, metastases are usually triggered, and the recovery rate will obviously be low, with extremely low chances of curing the patient. Therefore, in this last stage, the success rate with the use of resveratrol (as a type of treatment or adjuvant in treatment) cannot be ensured.

Combination therapy and the synergistic approach are and must be further studied through various research. As presented in the previous material, it has been found that the combined treatments have additionally improved effects with minimal side effects; moreover, it is obvious that the combination of drugs and resveratrol must be considered. The system of naturopathy and applying natural medicine also suggests that the consumption of herbs and natural ingredients is more active during administration for a certain time, considered optimal. If this point of view is clear, then the therapeutic management and effectiveness of the therapy components is maximized. Additionally, challenges and future perspectives in resveratrol availability for cancer therapy were also recently highlighted by Ren et al. [159]

The present review highlights new evidence of resveratrol as a preventive chemical agent and a conceptual basis for a new way to prevent and treat cancer. A more broad-based approach includes synergistic combinations of different low-toxic substances, such as micro-compounds (such as resveratrol plus other therapeutic compounds) or chemo drugs which may have possible implications for several well-known and important ways in cancer therapies, angiogenesis, and metastasis. The main protein involved in carcinogenesis may be p53, as a new emphasis in the study of translation, and will define possible ways of resveratrol influence.
